# Suicide mortality in the city of São Paulo: epidemiological characteristics and their social factors in a temporal trend between 2000 and 2017. Retrospective study

**DOI:** 10.1590/1516-3180.2019.0539.R1.05032020

**Published:** 2020-06-22

**Authors:** Patrícia Colombo-Souza, Fabio Boucault Tranchitella, Ana Paula Ribeiro, Yára Juliano, Neil Ferreira Novo

**Affiliations:** I PhD. Professor and Researcher, Postgraduate Department of Health Sciences, School of Medicine, Universidade de Santo Amaro (UNISA), São Paulo, Brazil.; II MD, MSc. Orthopedic Doctor, Postgraduate Department of Health Sciences, School of Medicine, Universidade de Santo Amaro (UNISA), São Paulo, Brazil.; III PhD. Professor and Coordinator, Biomechanics and Musculoskeletal Rehabilitation Laboratory, Postgraduate Department of Health Sciences, School of Medicine, Universidade de Santo Amaro (UNISA), São Paulo, Brazil; Postdoctoral Student, Department of Physical Therapy, Universidade de São Paulo (USP), São Paulo, Brazil.; IV MD. Professor and Researcher, Postgraduate Department of Health Sciences, School of Medicine, Universidade de Santo Amaro (UNISA), São Paulo, Brazil.; V MD. Professor and Researcher, Postgraduate Department of Health Sciences, Medical School, Universidade de Santo Amaro (UNISA), São Paulo, Brazil.

**Keywords:** Mortality, Suicide, Information systems, Epidemiology, Death, External causes, Social indicators, Suicide rates

## Abstract

**BACKGROUND:**

Suicide is one of the leading causes of death worldwide, accounting for one million deaths annually. Greater understanding of the causal risk factors is needed, especially in large urban centers.

**OBJECTIVE:**

To ascertain the epidemiological profile and temporal trend of suicides over two decades and correlate prevalence with social indicators.

**DESIGN AND SETTING:**

Descriptive population-based longitudinal retrospective study conducted in the city of São Paulo, Brazil.

**METHODS:**

A temporal trend series for suicide mortality in this city was constructed based on data from the Ministry of Health’s mortality notification system, covering 2000-2017. It was analyzed using classic demographic variables relating to social factors.

**RESULTS:**

Suicide rates were high throughout this period, increasing from 4.6/100,000 inhabitants in the 2000s to 4.9/100,000 in 2017 (mean: 4.7/100,000). The increase in mortality was mainly due to increased male suicide, which went from 6.0/100,000 to the current 8.0/100,000. Other higher coefficients corresponded to social risk factors, such as being a young adult (25-44 years old), being more educated (eight years of schooling) and having white ethnicity (67.2%). Suicide was also twice as likely to occur at home (47.8%).

**CONCLUSION:**

High suicide rates were seen over the period 2000-2017, especially among young adults and males. High schooling levels and white ethnicity were risk factors. The home environment is the crucial arena for preventive action. One special aspect of primary prevention is the internet and especially social media, which provides a multitude of information for suicide prevention.

## INTRODUCTION

Suicide is one of the three most common causes of death worldwide. The World Health Organization (WHO) has defined it as aggression or a violent act committed against one’s own life, with the intention of death.^1^ It is currently one of the most important public health problems and is often attributed to how its victims are affected by society and the collective environment in which they live.^2^ It has several supporting risk factors, such as psychological, biological and social factors.^3^

Suicide is among the 20 leading causes of death worldwide in different age groups, including adolescents, adults and the elderly. One death by suicide occurs every 40 seconds somewhere in the world.^4^ Findings from 2012 revealed that there were around 800,000 suicide deaths worldwide, representing an age-standardized overall annual rate of 11.4 per 100,000 inhabitants, within which males and the age group of young adults formed more significant components.^5^

In epidemiological studies, it has been estimated that by 2020, there will be a 50% increase in the annual incidence of suicide deaths worldwide, which could exceed the number of deaths from homicide and war.^6^^,^^7^ Suicide rates have undergone exponential increases in several countries such as France, China, Switzerland, Belgium, Austria, the United States, Japan and Brazil, as well as in countries in Eastern Europe, which report suicide rates above 16 deaths per 100,000 inhabitants.^6^^,^^7^ However, there is still great difficulty in assessing the dimensions of this problem and accurately recording suicidal acts.^8^

Globally, suicides are the second leading cause of premature mortality among individuals aged 15 to 29 years (preceded only by traffic accidents), and number three in the age group of 15-44 years.^9^ Upsettingly, in 2015, the vast majority, i.e. namely 78% of suicides, were committed in low and middle‐income countries (LMIC). High suicide rates also represent a financial burden to society, and this is most markedly so when young and middle-aged men who are about to start or have just started their professional and family lives commit suicide.^9^ In the United States, in the early 2010s, the costs per each single suicide were estimated to be over $1 million, while estimates from Ireland, Scotland and New Zealand lay between $2.1 and $2.5 million. In the literature, only 6% of the studies come from low-income countries low-income countries such as sub-Saharan Africa. Most studies have focused on measurements of poverty like unemployment and economic status, while neglecting dimensions such as debt, relative and absolute poverty, and support from welfare systems.^9^^,^^10^ This opens up a huge gap between the overall numbers of suicides in LMIC (78% of suicides worldwide) and knowledge of costs within the respective societies.^9^^,^^10^

Considering the significance that suicide has and the public health crisis that it has precipitated, a detailed understanding of the different age groups, genders, ethnicities and places with higher prevalence of suicide in large urban centers (where the characteristics of the environment increase the risks of mental disorders, such as depression, anxiety and stress) is essential. Through this, monitoring can be promoted and possible strategies for reducing individuals’ risk of suicide can be implemented.^11^^,^^12^^,^^13^

Although studies have shown that there has been a threefold increase in the suicide rate among males,^12^ there are still no reports of this rate over the course of two decades, or 18 consecutive years. Nor are there any reports on its prevalence in specific age groups, among specific ethnicities or in places in emerging countries with large urban populations. This phenomenon results from a complex network of biological, genetic, psychological, sociocultural and economic interactions.^13^

Studies have shown that suicide rates are increasing, but most studies have investigated only specific periods of time, or regions and states.^14^^,^^15^^,^^16^ However, greater specificity for actions and local preventive strategies is required.

## OBJECTIVES

The aims of this study were to analyze the epidemiological profile and temporal trends of suicide cases in the city of São Paulo, Brazil, over two decades, and to analyze the prevalence of these cases in relation to social indicators.

## METHODS

This was a mortality study (time series) that had the aim of characterizing aspects of suicide mortality in the city of São Paulo (SP), Brazil, from 2000 to 2017.

All the data used were obtained from official secondary sources. The number of suicides was obtained from the mortality information system (SIM) database and health information was obtained from a database (TABNET) maintained by the Brazilian Ministry of Health (DATASUS). The population of each state was obtained from the Brazilian Institute for Geography and Statistics (IBGE). Census data were taken from years in which censuses were performed, and were interpolated for the other years. These datasets are publicly available online.

Suicide was defined as death resulting from intentional self-harm, in accordance with the International Classification of Diseases, tenth edition (ICD-10), which uses codes X60 to X84 and Y87 to identify this outcome.

The 2000-2017 time series was composed of annual suicide rates. These were calculated as suicide mortality coefficients (using the numbers of occurrences divided by the general population per 100,000 inhabitants) and as standardized mortality (using the numbers of occurrences divided by the standardized population per 100,000 inhabitants) for the municipality of São Paulo. Analyses stratified according to sex, age group, education level, ethnicity and place where suicide was committed were also performed. Values corresponding to unknown age were excluded.

### Statistical analysis

We used the SPSS software, version 10.0, to identify and estimate suicide rates and standardized mortality coefficients. To calculate mortality coefficients, population data and data on mortality due to external causes were used. The overall mortality coefficient is one of the indicators of the state of health of a population. For the purposes of comparing populations, standardization was indispensable for correcting distortions resulting from possible differences in their composition with respect to attributes or variables related to the probability of death.^17^

The median of the participation percentages, rather than the arithmetic mean, was used with the aim of removing the influence that, in this case, would be exerted by possible occurrences, in certain populations, of strongly disagreeing percentages of participation.^18^ The denominator that was used to calculate the standardized coefficient was that of the standard population, which was calculated using the median population of the period studied. Historical series were built for the period from 2000 to 2017.

## RESULTS

Over the 18 years that made up this analysis, there were 8,726 deaths due to suicide in the city of São Paulo, corresponding to 4.7 deaths per 100,000 inhabitants, while the world averages ranged from 3.5 to 4.0 deaths per 100,000 inhabitants.

The coefficients were standardized using the standard population provided by the World Health Organization (WHO). The gross coefficient of the population increased from 4.08/100,000 in 2000 to 4.69/100,000 in 2017. It also needs to be taken into account that, because of the taboo surrounding suicide, deaths due to this event may be reported as deaths due to an external cause of unknown type. This may have induced underreporting of the problem.

Table 1 shows the original and standardized suicide mortality rates for the municipality of São Paulo in this time series from 2000 to 2017. The standardized suicide mortality rates for both sexes increased from 3.84/100.000 in 2000 to 4.96/100.000 in 2017. The increase in the male standardized coefficient was from 6.08/100.000 in 2000 to 8.09/100.000 in 2017, while for females it increased from 1.91 to 1.97/100,000 over this same period. The male-to-female ratio increased from four in 2000 to six at the end of the study period, in 2017. The temporal trend showed that higher male rates were maintained throughout the study period, without any abrupt increase.

**Table 1.** Mortality coefficients relating to crude and standardized suicide, according to sex and total data. São Paulo (SP), 2000-2017

Years	Death (male)	Male sex coefficient Mortality suicide		Death (female)	Female sex coefficient Mortality suicide		Death (All)	Coefficient mortality suicide	
		Original	Standardized		Original	Standardized		Original	Standardized
2000	321	6.46	6.08	104	1.91	1.78	425	4.08	3.84
2001	311	6.20	5.89	107	1.94	1.83	418	3.97	3.78
2002	305	6.04	5.78	90	1.62	1.54	395	3.72	3.57
2003	321	6.31	6.08	94	1.68	1.61	415	3.88	3.75
2004	303	5.91	5.74	97	1.72	1.66	400	3.71	3.62
2005	334	6.47	6.33	121	2.12	2.07	455	4.19	4.12
2006	376	7.23	7.13	94	1.64	1.61	470	4.29	4.25
2007	379	7.25	7.18	113	1.95	1.93	492	4.46	4.45
2008	392	7.45	7.43	106	1.82	1.81	498	4.49	4.50
2009	394	7.44	7.47	122	2.08	2.08	516	4.62	4.67
2010	400	7.51	7.58	130	2.20	2.22	530	4.71	4.79
2011	409	7.63	7.75	129	2.17	2.20	538	4.76	4.87
2012	407	7.54	7.71	154	2.57	2.63	561	4.93	5.07
2013	396	7.29	7.50	146	2.43	2.49	542	4.74	4.90
2014	401	7.34	7.60	133	2.20	2.27	534	4.64	4.83
2015	388	7.05	7.35	140	2.30	2.39	528	4.56	4.78
2016	365	6.60	6.92	96	1.57	1.64	461	3.96	4.17
2017	427	7.68	8.09	121	1.97	2.07	548	4.69	4.96
Total	6,629	6.98	6.97	2,097	2.00	1.99	8,726	4.70	4.97

Table 2 shows the numbers of deaths, proportional mortality and suicide mortality coefficient according to age, education, ethnicity and place of suicide, in the 18 consecutive years of the period from 2000 to 2017. Higher proportions of suicides occurred among young adults (25 to 44 years old) and individuals with higher education (more than 8 years of schooling). However, the highest coefficient was for individuals of white ethnicity (67.2%) and for suicide occurrences at home (47.8%). The risk in the white population (2.91/100.000) was almost three times higher than in the other populations. In addition, another important finding was that the chance of suicide occurring at home was twice as high (2.07/100,000) as in other places, over the years.

**Table 2.** Proportional mortality due to suicide and standardized mortality rates for suicide in São Paulo (SP) (2000-2017), according to sociodemographic characteristics (age, education, ethnicity and place of occurrence)

Sociodemographic characteristics	Proportional mortality		Standardized coefficient
	n	%	
Age range (years)			
5-14	73	0.8	0.03
15-24	1,501	17.3	0.75
25-34	2,145	24.7	1.07
35-44	1,848	21.2	0.92
45-54	1,394	16.1	0.69
55-64	878	10.2	0.43
65-74	460	5.3	0.23
75 and more	381	4.4	0.19
Total	8,680*	100.0	4.34
Schooling level (years)			
None	139	1.7	0.07
1 to 3	906	11.2	0.45
4 to 7	2,657	33.1	1.32
8 to 11	2,874	35.7	1.43
12 and more	1,466	18.3	0.73
Total	8,042**	100.0	4.02
Ethnicity			
White	5,821	67.2	2.91
Black	487	5.6	0.24
East Asian	165	1.9	0.08
Brown	2,196	25.3	1.09
Indigenous	3	0.03	0.001
Total	8,672***	100.0	4.33
Place of occurrence			
Hospital	2,866	32.8	1.43
Home	4,157	47.8	2.07
Public highway	622	7.2	0.31
Healthcare centers	44	0.5	0.02
Others	1,015	11.7	0.50
Total	8,704****	100.0	4.35

*46 without age information; **684 without education information; ***54 without ethnicity information; ****22 without location information.

## DISCUSSION

The main objective of this study was to analyze the epidemiological profile and temporal trends of suicide cases in the city of São Paulo over two decades and to analyze the prevalence of suicide in relation to social indicators. The main finding was that there was a great number of deaths (8,726) due to suicide in the municipality studied, in comparison with other countries, corresponding to 4.7 deaths per 100,000 inhabitants, while the world average ranged from 3.5 to 4.0 deaths per 100,000 inhabitants. Another important finding was that there was an increase in the male standardized coefficient from 6.08/100,000 in 2000 to 8.09/100,000 in 2017. Regarding social indicators, there were higher proportions of suicides among young adults (25-44 years), individuals with higher education and individuals of white ethnicity. The place where most suicides were committed was the home, which was twice as high as in other places, over the years.

In Brazil, the average mortality rate due to suicide over the period 2004-2010 was 5.7%.^19^^,^^20^ Over the last two decades (2000-2017), this rate was lower in the city of São Paulo (4.7), but compared with other countries, it can still be considered high and of significant impact. The world average ranged from 3.5 to 4.0, and the coefficients found in European countries were at this average level. The lowest coefficients were in Central and South American countries, while the coefficients in the United States, Australia, Japan and Central European countries were in an intermediate range.^14^^,^^21^

The differential of the present study was that a suicide rate of 4.7 was ascertained in a single municipality. This urban center can be considered to be a reference point, given its large population. The results from this city showed that there is a need for greater support for preventive and management actions within public policies.

Another important point observed in this study was that the suicide mortality rate was higher among males (standardized coefficient of 8.09/100,000). Thus, men committed suicide almost twice as often as women in the city of São Paulo, Brazil, over the 18-year period 2000-2017. However, comparison of information from 183 countries shows that the male-to-female ratio varies.^9^ Several studies have documented the epidemiology of higher suicide rates among males, which in some countries have reached a ratio of 3:1.^14^^,^^19^ Higher rates have also been observed in small and medium-sized cities and municipalities.^20^^,^^22^ One exception is India and China, where the suicide rate among females exceeds that of males.^23^

The findings of the present study showed that there was higher prevalence of suicide among males than among females. The explanation for this, according to some authors,^24^^,^^25^^,^^26^ relates to manifestations of masculinity, which involves behaviors that predispose towards suicide, such as competitiveness, impulsivity and greater access to lethal weapons, including firearms.

In addition, failure to fulfill traditional gender roles, which for men means being the economic provider for the family, generates greater stress and anxiety. Men who live within a patriarchal culture are more sensitive to economic setbacks such as unemployment and impoverishment and more prone to suicide.^25^ This was corroborated by the higher rates and temporal trends seen among men in this large-sized city, over the 18 years evaluated in the present study. The lower prevalence among women can perhaps be attributed to lower prevalence of alcoholism, religiosity and flexible attitudes towards social skills and role-playing over their lifetimes.^27^

Another finding of great relevance for public health was the social indicators ascertained in this study. It was observed that young adults, aged between 25 and 44 years, individuals with higher education and individuals of white ethnicity presented higher prevalence of suicide. The place where suicide was committed most effectively was at home, and this proportion was twice as high as in other places, over the years. This can clearly be explained by the high rates of suicide that exist among young adults in low-income countries, due to inequality. Low quality of healthcare and poor access to it most likely play a role.

Some studies have shown that the adolescent and young adult population (between 20 and 50 years old) has experienced a significant increase in suicide rates.^28^^,^^29^ Nevertheless, the elderly population (over 60 years old) still has the highest absolute rates, compared with the world average, and higher rates than in the population between 20 and 59 years old.^8^^,^^27^^,^^28^^,^^29^^,^^30^ On the other hand, in the present study, the highest prevalence remained among adolescents and young adults. This may be explained by the higher work overloads and poorer quality of life in large urban population centers, as is the case of São Paulo, Brazil.

The higher suicide rates among young adults, individuals with higher education and individuals of white ethnicity forms an alarming scenario. It provides clear evidence that ethnicity, country of origin and country of settlement influence the risk of suicide, not least because cultural differences between countries may cause intergenerational and intrapsychic conflicts. So far, there is little evidence in the literature, from specific studies on factors that might be considered to be risk factors for suicide.^16^ Higher schooling levels and white ethnicity were significant social factors in the city of São Paulo, for trigger suicidal actions, and preventive measures directed towards this segment of the population need to be implemented public.

The place of the suicidal act has been well discussed in the literature. The risk of suicide is around three to five times higher in hospital environments than in the population as a whole.^31^ Most hospital-related cases are associated with chronic or terminal illnesses that have become painful and debilitating.^31^^,^^32^ However, in the present study, the opposite was observed, i.e. the prevalence of suicide was twice as high, over the 18-year period, in the home environment. Across the world, there is little data on the places where suicide attempts are made. If present, the quality of such data is low due to a lack of reliable statistics, which relates to underdiagnosis, misdiagnosis or non-diagnosis and reporting. The WHO does not receive information from any country in the world on this topic, although at least data from emergency rooms and somatic hospitals might be obtained, along with some self-reports. The results from the present study corroborate the data of Lovisi et al.,^14^ which showed that the home was the most frequent scenario for suicides, accounting for 51%, followed by hospitals, with 26%. The predominant means used were hanging (47%), firearms (19%) and poisoning (14%).^14^

The limitation of the present study was that the real degrees of underestimation and underreporting of data were not ascertained. Thus, the true prevalence of suicide may have been greater, considering the extreme difficulty in accurately assessing the scale of suicidal acts and recording them.

## CONCLUSION

Information from the city of São Paulo covering the years 2000-2017 led to the perception that suicide rates were high, with proportions of 4.7/100,000 inhabitants, and with high prevalence among young adults and males. The risk factors influencing high suicide rates were high schooling levels and white ethnicity. The crucial factor in successfully committing suicide was the home environment. One special aspect of primary prevention relates to the internet and especially social media, which provide a multitude of information for suicide prevention.
